# Crystal structure of ethyl (2*S*)-9-meth­oxy-2-methyl-4-oxo-3,4,5,6-tetra­hydro-2*H*- 2,6-methano­benzo[*g*][1,3,5]oxa­diazocine-11-carboxyl­ate

**DOI:** 10.1107/S2056989015000559

**Published:** 2015-01-17

**Authors:** A. Dhandapani, S. Manivarman, S. Subashchandrabose, B. Gunasekaran

**Affiliations:** a Post Graduate and Research Department of Chemistry, Government Arts College, C-Mutlur, Chidambaram 608 102, Tamil Nadu, India; b Centre for Research & Development, PRIST University, Vallam, Thanjavur 613 403, Tamil Nadu, India; c Department of Physics & Nano Technology, SRM University, SRM Nagar, Kattankulathur, Kancheepuram Dist, Chennai 603 203, Tamil Nadu, India

**Keywords:** crystal structure, hydro­pyrimidine, oxa­diazo­cine, pyran, hydrogen bonding

## Abstract

In the title compound, C_15_H_18_N_2_O_5_, the meth­oxy­phenyl ring makes a dihedral angle of 84.70 (12)° with the mean plane of the tetra­hydro­pyrimidin-2(1*H*)-one ring. Both the pyran and tetra­hydro­pyrimidin-2(1*H*)-one rings have distorted envelope conformations with the carboxyl­ate-substituted C atom as the flap. In the crystal, mol­ecules are linked *via* pairs of N—H⋯O hydrogen bonds, forming zigzag chains propagating along [010], which enclose *R* ^2^_2_(8) ring motifs. The chains are linked by C—H⋯π inter­actions, forming a two-dimensional network parallel to (100).

## Related literature   

For the biological activity of di­hydro­pyrimidine derivatives, see: Hurst & Hull (1961 ▸); Ashok *et al.* (2007 ▸); Bahekar & Shinde (2004 ▸); Mayer *et al.* (1999 ▸); Kappe (2000 ▸); For the crystal structures of two very similar compounds, see: Jing *et al.* (2009 ▸); Yar *et al.* (2014 ▸; Liu *et al.* (2014 ▸).
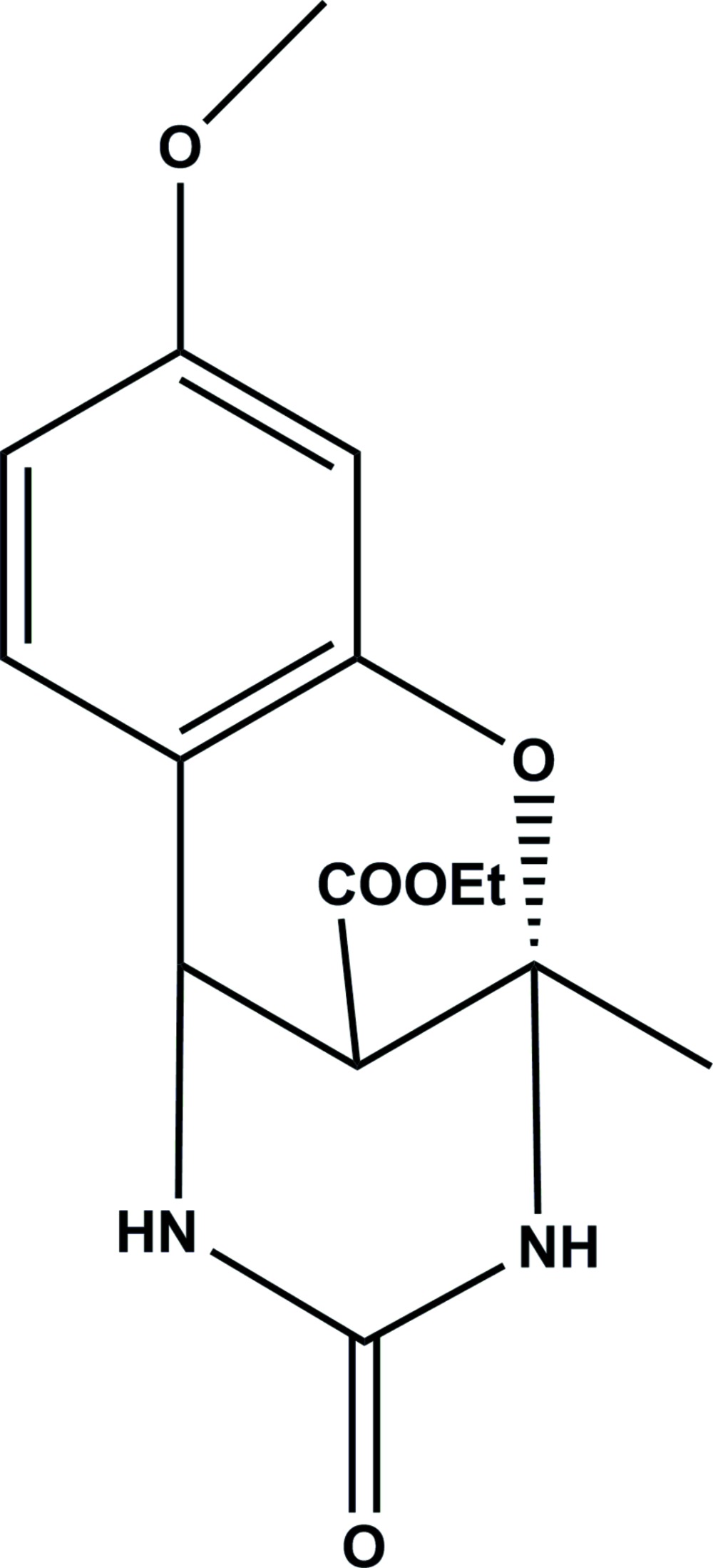


## Experimental   

### Crystal data  

C_15_H_18_N_2_O_5_
*M* *_r_* = 306.31
Monoclinic, 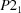

*a* = 9.6982 (14) Å
*b* = 7.4802 (12) Å
*c* = 10.8293 (17) Å
β = 111.252 (5)°
*V* = 732.2 (2) Å^3^
*Z* = 2
Mo *K*α radiation
μ = 0.11 mm^−1^
*T* = 295 K
0.25 × 0.20 × 0.20 mm

### Data collection  

Bruker APEXII CCD diffractometer
Absorption correction: multi-scan (*SADABS*; Sheldrick, 1996 ▸) *T*_min_ = 0.954, *T*_max_ = 0.975
10886 measured reflections
3030 independent reflections
2029 reflections with *I* > 2σ(*I*)
*R*_int_ = 0.053

### Refinement  

*R*[*F* ^2^ > 2σ(*F* ^2^)] = 0.071
*wR*(*F* ^2^) = 0.175
*S* = 1.14
3030 reflections
203 parameters
1 restraint
H-atom parameters constrained
Δρ_max_ = 0.26 e Å^−3^
Δρ_min_ = −0.25 e Å^−3^
Absolute structure: Flack (1983 ▸), 1371 Friedel pairs
Absolute structure parameter: 0.01 (4)

### 

Data collection: *APEX2* (Bruker, 2008 ▸); cell refinement: *SAINT* (Bruker, 2008 ▸); data reduction: *SAINT*; program(s) used to solve structure: *SHELXS97* (Sheldrick, 2008 ▸); program(s) used to refine structure: *SHELXL97* (Sheldrick, 2008 ▸, 2015 ▸); molecular graphics: *PLATON* (Spek, 2009 ▸); software used to prepare material for publication: *SHELXL97*.

## Supplementary Material

Crystal structure: contains datablock(s) I. DOI: 10.1107/S2056989015000559/su5057sup1.cif

Structure factors: contains datablock(s) I. DOI: 10.1107/S2056989015000559/su5057Isup2.hkl

Supporting information file. DOI: 10.1107/S2056989015000559/su5057Isup3.cml

**Fig. 1.**
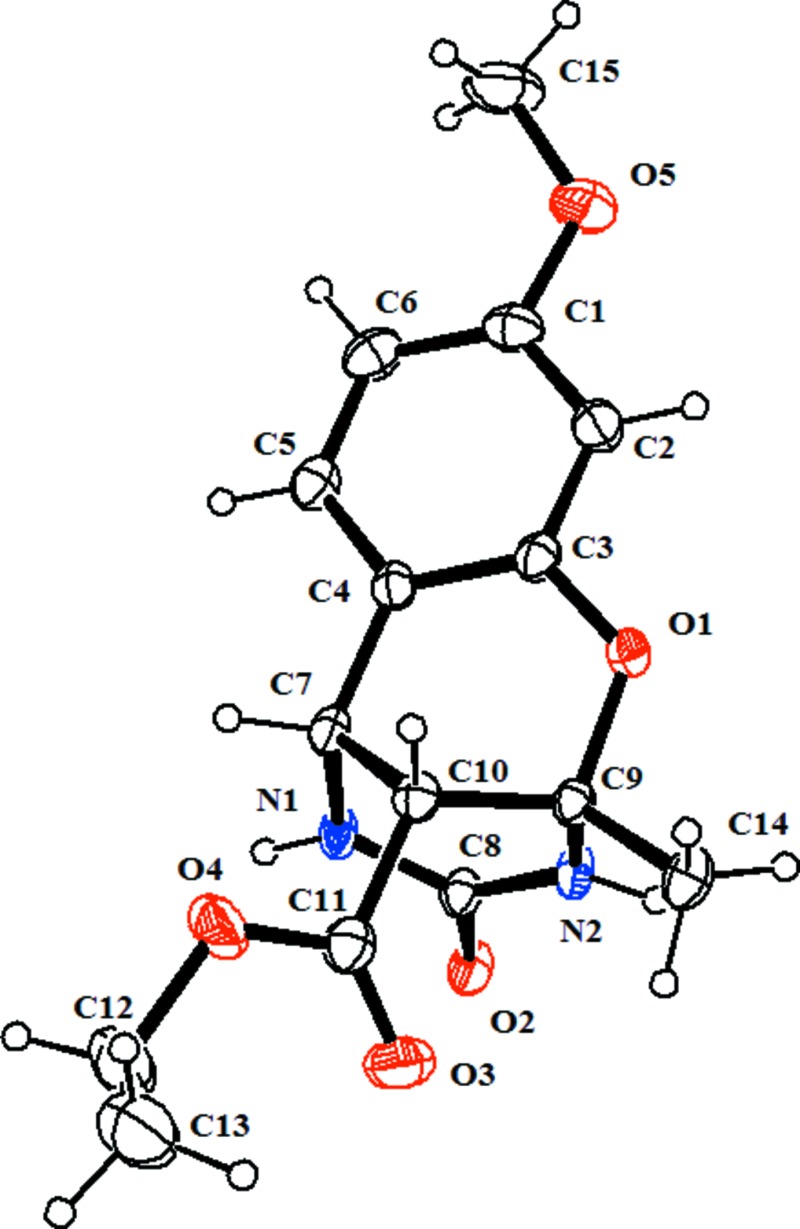
The molecular structure of the title compound, with atom labelling. Displacement ellipsoids are drawn at the 30% probability level.

**Fig. 2.**
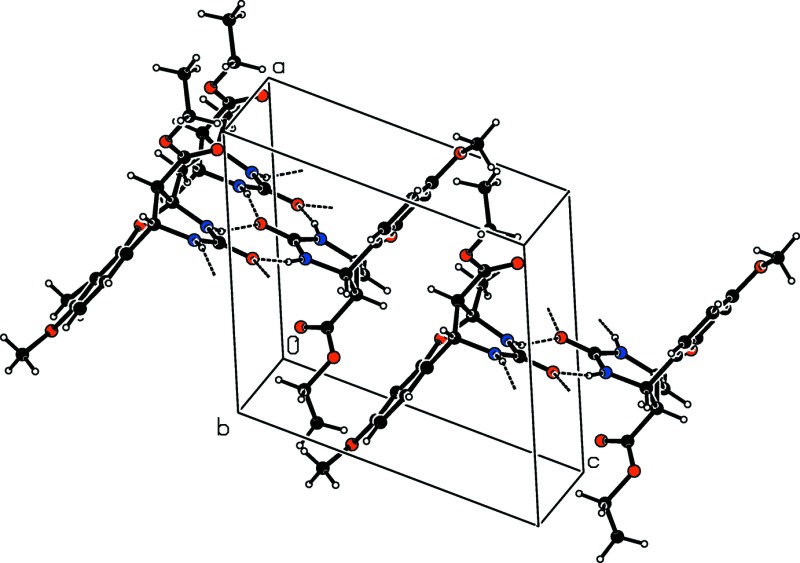
The crystal packing of the title compound, viewed along the b axis. Hydrogen bonds are shown as dashed lines (see Table 1 for details).

. DOI: 10.1107/S2056989015000559/su5057fig1.tif
The mol­ecular structure of the title compound, with atom labelling. Displacement ellipsoids are drawn at the 30% probability level.

b . DOI: 10.1107/S2056989015000559/su5057fig2.tif
The crystal packing of the title compound, viewed along the *b* axis. Hydrogen bonds are shown as dashed lines (see Table 1 for details).

CCDC reference: 1043105

Additional supporting information:  crystallographic information; 3D view; checkCIF report

## Figures and Tables

**Table 1:** Hydrogen-bond geometry (, ) *Cg*1 is the centroid of the C1C6 ring.

*D*H*A*	*D*H	H*A*	*D* *A*	*D*H*A*
N1H1O2^i^	0.86	2.12	2.962(5)	168
N2H2*A*O2^ii^	0.86	2.11	2.936(4)	162
C14H14*B* *Cg*1^iii^	0.96	2.62	3.570(6)	171

Symmetry codes: (i) 
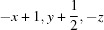
; (ii) 
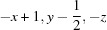
; (iii) 
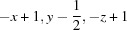
.

## supplementary crystallographic information

### S1. Comment

Dihydropyrimidine derivatives have recently received great attention because of their wide range of therapeutic and pharmacological properties, such as antiviral (Hurst & Hull, 1961), antitumor, antibacterial and antifungal (Ashok *et al.*, 2007), anti-inflammatory (Bahekar & Shinde, 2004), antihypertensive agents, and neuropeptide Y (NPY) antagonists (Mayer *et al.*, 1999). The natural products containing these heterocyclic moieties have been studied as new leads for AIDS therapies (Kappe, 2000).

The molecular structure of the title compound is illustrated in Fig. 1. The methoxyphenyl ring (C1-C6) makes a dihedral angles of 84.70 (12) ° with the mean plane of the tetrahydropyrimidin-2(1*H*)-one ring (N1/N2/C7-C10). Both the pyran (O1/C3/C4/C7/C10)and tetrahydropyrimidin-2(1*H*)-one rings have distorted envelope con formations with atom C10 as the flap.

The geometrical parameters of the title molecule agree well with those reported for two very similar compounds, *viz.* ethyl 2-methyl-4-oxo-3,4,5,6-tetrahydro-2*H*-2,6-methanobenzo[*g*][1,3,5]oxadiazocine-11-carboxylate (Jing *et al.*, 2009; Yar *et al.*, 2014) and methyl 2-methyl-4-oxo-3,4,5,6-tetrahydro-2*H*-2,6-methanobenzo[*g*][1,3,5]oxadiazocine-11-carboxylate (Liu *et al.*, 2014)

In the crystal, molecules are linked via pairs of N—H···O hydrogen bonds forming zigzag chains propagating along [010], which enclose R^2^_2_(8) ring motifs (Table 1 and Fig. 2). The chains are linked by C-H···π interactions forming a two-dimensional network parallel to (100).

### S2. Experimental

2-hydroxy-4-methoxybenzaldehyde (0.76 g, 5 mmol) and urea (0.9 g, 15 mmol) were added to an ethanolic solution of ethyl acetoacetate (0.65 ml, 5 mmol). To this mixture CeCl_3_.7H_2_O (0.465 g, 25%) was added slowly with stirring. After the addition was complete the reaction mixture was reflux at 363 K. The reaction mixture was then cooled to room temperature, then poured onto crushed ice water and stirred for 10 min. The solid that separated was filtered under suction, washed with water, and then recrystallized from DMSO giving colourless block-like crystals (yield: 96%; m.p.: 463 K).

### S3. Refinement

H atoms were positioned geometrically and refined using a riding model: N-H = 0.86 Å, C—H = 0.93 - 98 Å with *U*_iso_(H) = 1.5U_eq_(C) for methyl H atoms and = 1.2U_eq_(N,C) for other H atoms.

### Crystal data

**Table:**

C_15_H_18_N_2_O_5_	*F*(000) = 324
*M**_r_* = 306.31	*D*_x_ = 1.389 Mg m^−^^3^
Monoclinic, *P*2_1_	Mo *K*α radiation, λ = 0.71073 Å
Hall symbol: P 2yb	Cell parameters from 3030 reflections
*a* = 9.6982 (14) Å	θ = 2.0–26.8°
*b* = 7.4802 (12) Å	µ = 0.11 mm^−^^1^
*c* = 10.8293 (17) Å	*T* = 295 K
β = 111.252 (5)°	Block, colourless
*V* = 732.2 (2) Å^3^	0.25 × 0.20 × 0.20 mm
*Z* = 2	

### Data collection

**Table:**

Bruker APEXII CCD diffractometer	3030 independent reflections
Radiation source: fine-focus sealed tube	2029 reflections with *I* > 2σ(*I*)
Graphite monochromator	*R*_int_ = 0.053
Detector resolution: 0 pixels mm^-1^	θ_max_ = 26.9°, θ_min_ = 2.0°
ω and φ scans	*h* = −11→12
Absorption correction: multi-scan (*SADABS*; Sheldrick, 1996)	*k* = −9→9
*T*_min_ = 0.954, *T*_max_ = 0.975	*l* = −13→13
10886 measured reflections	

### Refinement

**Table:**

Refinement on *F*^2^	Hydrogen site location: inferred from neighbouring sites
Least-squares matrix: full	H-atom parameters constrained
*R*[*F*^2^ > 2σ(*F*^2^)] = 0.071	*w* = 1/[σ^2^(*F*_o_^2^) + (0.0537*P*)^2^ + 0.7506*P*] where *P* = (*F*_o_^2^ + 2*F*_c_^2^)/3
*wR*(*F*^2^) = 0.175	(Δ/σ)_max_ < 0.001
*S* = 1.14	Δρ_max_ = 0.26 e Å^−^^3^
3030 reflections	Δρ_min_ = −0.25 e Å^−^^3^
203 parameters	Extinction correction: *SHELXL97* (Sheldrick, 2008)
1 restraint	Extinction coefficient: 0.031 (8)
Primary atom site location: structure-invariant direct methods	Absolute structure: Flack (1983), 1371 Friedel pairs
Secondary atom site location: difference Fourier map	Absolute structure parameter: 0.01 (4)

### Fractional atomic coordinates and isotropic or equivalent isotropic displacement parameters (Å^2^)

**Table:**

	*x*	*y*	*z*	*U*_iso_*/*U*_eq_	
C1	0.9053 (5)	0.2379 (7)	0.5750 (5)	0.0437 (13)	
C2	0.8132 (5)	0.0956 (7)	0.5239 (5)	0.0412 (12)	
H2	0.8430	−0.0198	0.5535	0.049*	
C3	0.6753 (5)	0.1257 (6)	0.4278 (4)	0.0325 (10)	
C4	0.6293 (5)	0.2978 (6)	0.3837 (4)	0.0309 (10)	
C5	0.7245 (6)	0.4362 (6)	0.4375 (5)	0.0375 (11)	
H5	0.6948	0.5519	0.4085	0.045*	
C6	0.8629 (6)	0.4105 (7)	0.5331 (5)	0.0455 (13)	
H6	0.9257	0.5066	0.5682	0.055*	
C7	0.4765 (5)	0.3254 (6)	0.2807 (4)	0.0299 (10)	
H7	0.4353	0.4379	0.2984	0.036*	
C8	0.4926 (4)	0.1772 (6)	0.0871 (4)	0.0302 (9)	
C9	0.4544 (5)	0.0033 (6)	0.2670 (4)	0.0295 (10)	
C10	0.3772 (4)	0.1706 (6)	0.2878 (4)	0.0308 (9)	
H10	0.3722	0.1671	0.3765	0.037*	
C11	0.2243 (5)	0.2001 (7)	0.1873 (5)	0.0412 (11)	
C12	0.0178 (7)	0.3958 (9)	0.1308 (6)	0.0700 (19)	
H12A	0.0121	0.3701	0.0413	0.084*	
H12B	0.0123	0.5244	0.1396	0.084*	
C13	−0.1071 (7)	0.3110 (11)	0.1540 (7)	0.088 (2)	
H13A	−0.1119	0.1870	0.1299	0.132*	
H13B	−0.1974	0.3693	0.1011	0.132*	
H13C	−0.0937	0.3212	0.2460	0.132*	
C14	0.3703 (5)	−0.1689 (6)	0.2594 (5)	0.0398 (12)	
H14A	0.2826	−0.1685	0.1810	0.060*	
H14B	0.3432	−0.1792	0.3361	0.060*	
H14C	0.4316	−0.2683	0.2564	0.060*	
C15	1.1301 (7)	0.3274 (10)	0.7503 (6)	0.074 (2)	
H15A	1.1513	0.4157	0.6953	0.111*	
H15B	1.2209	0.2756	0.8085	0.111*	
H15C	1.0794	0.3828	0.8017	0.111*	
N1	0.4778 (4)	0.3287 (5)	0.1464 (3)	0.0341 (9)	
H1	0.4690	0.4288	0.1051	0.041*	
N2	0.4908 (4)	0.0226 (4)	0.1509 (3)	0.0326 (9)	
H2A	0.5136	−0.0733	0.1187	0.039*	
O1	0.5875 (3)	−0.0211 (4)	0.3833 (3)	0.0343 (8)	
O2	0.5039 (3)	0.1774 (4)	−0.0236 (3)	0.0383 (8)	
O3	0.1709 (4)	0.1240 (6)	0.0862 (4)	0.0817 (15)	
O4	0.1593 (4)	0.3304 (6)	0.2256 (4)	0.0650 (12)	
O5	1.0392 (4)	0.1917 (6)	0.6693 (4)	0.0682 (12)	

### Atomic displacement parameters (Å^2^)

**Table:**

	*U*^11^	*U*^22^	*U*^33^	*U*^12^	*U*^13^	*U*^23^
C1	0.033 (3)	0.059 (3)	0.037 (3)	0.002 (2)	0.010 (2)	−0.003 (2)
C2	0.044 (3)	0.040 (3)	0.039 (3)	0.006 (2)	0.014 (2)	0.003 (2)
C3	0.040 (3)	0.028 (2)	0.031 (2)	−0.001 (2)	0.016 (2)	−0.0004 (18)
C4	0.039 (3)	0.028 (2)	0.028 (2)	0.003 (2)	0.015 (2)	0.0012 (19)
C5	0.046 (3)	0.033 (3)	0.038 (3)	−0.003 (2)	0.020 (2)	0.000 (2)
C6	0.046 (3)	0.044 (3)	0.048 (3)	−0.015 (3)	0.018 (3)	−0.008 (2)
C7	0.042 (3)	0.023 (2)	0.028 (2)	0.000 (2)	0.017 (2)	−0.0006 (18)
C8	0.039 (2)	0.026 (2)	0.029 (2)	0.001 (2)	0.0161 (18)	0.000 (2)
C9	0.037 (2)	0.025 (2)	0.028 (2)	0.000 (2)	0.013 (2)	0.0020 (18)
C10	0.036 (2)	0.031 (2)	0.030 (2)	0.001 (2)	0.0173 (18)	−0.002 (2)
C11	0.042 (3)	0.040 (3)	0.042 (3)	0.002 (2)	0.016 (2)	−0.004 (2)
C12	0.060 (4)	0.077 (4)	0.065 (4)	0.028 (4)	0.014 (3)	0.009 (3)
C13	0.060 (4)	0.084 (5)	0.099 (5)	0.006 (4)	0.003 (4)	0.011 (5)
C14	0.051 (3)	0.030 (2)	0.047 (3)	−0.007 (2)	0.028 (2)	−0.002 (2)
C15	0.057 (4)	0.089 (5)	0.055 (3)	−0.020 (4)	−0.006 (3)	0.004 (4)
N1	0.058 (3)	0.0218 (18)	0.0261 (19)	0.0069 (18)	0.0197 (18)	0.0055 (15)
N2	0.050 (2)	0.0202 (19)	0.036 (2)	0.0038 (17)	0.0244 (19)	0.0013 (15)
O1	0.0418 (18)	0.0264 (16)	0.0315 (16)	0.0019 (14)	0.0092 (14)	0.0033 (13)
O2	0.062 (2)	0.0286 (16)	0.0309 (15)	−0.0006 (17)	0.0244 (14)	0.0015 (14)
O3	0.058 (3)	0.089 (3)	0.071 (3)	0.019 (2)	−0.011 (2)	−0.041 (3)
O4	0.060 (2)	0.074 (3)	0.051 (2)	0.037 (2)	0.0092 (19)	−0.004 (2)
O5	0.044 (2)	0.073 (3)	0.066 (2)	0.007 (2)	−0.0064 (19)	−0.008 (2)

### Geometric parameters (Å, º)

**Table:**

C1—C2	1.371 (7)	C9—C10	1.516 (6)
C1—O5	1.373 (6)	C10—C11	1.504 (6)
C1—C6	1.381 (7)	C10—H10	0.9800
C2—C3	1.383 (6)	C11—O3	1.174 (5)
C2—H2	0.9300	C11—O4	1.308 (6)
C3—O1	1.366 (5)	C12—O4	1.468 (7)
C3—C4	1.389 (6)	C12—C13	1.468 (9)
C4—C5	1.369 (6)	C12—H12A	0.9700
C4—C7	1.511 (6)	C12—H12B	0.9700
C5—C6	1.379 (7)	C13—H13A	0.9600
C5—H5	0.9300	C13—H13B	0.9600
C6—H6	0.9300	C13—H13C	0.9600
C7—N1	1.459 (5)	C14—H14A	0.9600
C7—C10	1.526 (6)	C14—H14B	0.9600
C7—H7	0.9800	C14—H14C	0.9600
C8—O2	1.242 (5)	C15—O5	1.420 (7)
C8—N1	1.336 (5)	C15—H15A	0.9600
C8—N2	1.351 (5)	C15—H15B	0.9600
C9—N2	1.431 (6)	C15—H15C	0.9600
C9—O1	1.452 (5)	N1—H1	0.8600
C9—C14	1.510 (6)	N2—H2A	0.8600
			
C2—C1—O5	114.0 (5)	C7—C10—H10	109.1
C2—C1—C6	121.3 (4)	O3—C11—O4	123.8 (5)
O5—C1—C6	124.6 (5)	O3—C11—C10	126.1 (4)
C1—C2—C3	119.3 (4)	O4—C11—C10	110.0 (4)
C1—C2—H2	120.4	O4—C12—C13	110.9 (5)
C3—C2—H2	120.4	O4—C12—H12A	109.5
O1—C3—C2	116.3 (4)	C13—C12—H12A	109.5
O1—C3—C4	122.9 (4)	O4—C12—H12B	109.5
C2—C3—C4	120.8 (4)	C13—C12—H12B	109.5
C5—C4—C3	118.1 (4)	H12A—C12—H12B	108.1
C5—C4—C7	122.8 (4)	C12—C13—H13A	109.5
C3—C4—C7	119.1 (4)	C12—C13—H13B	109.5
C4—C5—C6	122.5 (4)	H13A—C13—H13B	109.5
C4—C5—H5	118.8	C12—C13—H13C	109.5
C6—C5—H5	118.8	H13A—C13—H13C	109.5
C5—C6—C1	118.1 (5)	H13B—C13—H13C	109.5
C5—C6—H6	121.0	C9—C14—H14A	109.5
C1—C6—H6	121.0	C9—C14—H14B	109.5
N1—C7—C4	112.2 (4)	H14A—C14—H14B	109.5
N1—C7—C10	107.3 (3)	C9—C14—H14C	109.5
C4—C7—C10	109.1 (3)	H14A—C14—H14C	109.5
N1—C7—H7	109.4	H14B—C14—H14C	109.5
C4—C7—H7	109.4	O5—C15—H15A	109.5
C10—C7—H7	109.4	O5—C15—H15B	109.5
O2—C8—N1	121.7 (4)	H15A—C15—H15B	109.5
O2—C8—N2	121.1 (4)	O5—C15—H15C	109.5
N1—C8—N2	117.2 (3)	H15A—C15—H15C	109.5
N2—C9—O1	110.5 (3)	H15B—C15—H15C	109.5
N2—C9—C14	109.8 (3)	C8—N1—C7	120.4 (4)
O1—C9—C14	103.5 (3)	C8—N1—H1	119.8
N2—C9—C10	109.8 (3)	C7—N1—H1	119.8
O1—C9—C10	107.8 (3)	C8—N2—C9	126.1 (3)
C14—C9—C10	115.2 (4)	C8—N2—H2A	117.0
C11—C10—C9	115.1 (4)	C9—N2—H2A	117.0
C11—C10—C7	109.1 (4)	C3—O1—C9	116.7 (3)
C9—C10—C7	105.3 (3)	C11—O4—C12	117.5 (4)
C11—C10—H10	109.1	C1—O5—C15	119.1 (5)
C9—C10—H10	109.1		
			
O5—C1—C2—C3	−179.8 (4)	N1—C7—C10—C9	64.6 (4)
C6—C1—C2—C3	0.4 (7)	C4—C7—C10—C9	−57.2 (4)
C1—C2—C3—O1	−178.5 (4)	C9—C10—C11—O3	−12.8 (7)
C1—C2—C3—C4	−0.5 (7)	C7—C10—C11—O3	105.3 (6)
O1—C3—C4—C5	178.2 (4)	C9—C10—C11—O4	170.0 (4)
C2—C3—C4—C5	0.4 (6)	C7—C10—C11—O4	−71.9 (5)
O1—C3—C4—C7	−0.9 (6)	O2—C8—N1—C7	−175.1 (4)
C2—C3—C4—C7	−178.7 (4)	N2—C8—N1—C7	6.6 (6)
C3—C4—C5—C6	−0.2 (7)	C4—C7—N1—C8	75.5 (5)
C7—C4—C5—C6	178.9 (4)	C10—C7—N1—C8	−44.2 (5)
C4—C5—C6—C1	0.0 (7)	O2—C8—N2—C9	−169.1 (4)
C2—C1—C6—C5	−0.2 (7)	N1—C8—N2—C9	9.1 (6)
O5—C1—C6—C5	−179.9 (5)	O1—C9—N2—C8	−103.2 (5)
C5—C4—C7—N1	87.0 (5)	C14—C9—N2—C8	143.3 (4)
C3—C4—C7—N1	−94.0 (5)	C10—C9—N2—C8	15.7 (6)
C5—C4—C7—C10	−154.3 (4)	C2—C3—O1—C9	−169.5 (4)
C3—C4—C7—C10	24.7 (5)	C4—C3—O1—C9	12.6 (6)
N2—C9—C10—C11	69.2 (5)	N2—C9—O1—C3	72.7 (4)
O1—C9—C10—C11	−170.3 (3)	C14—C9—O1—C3	−169.9 (4)
C14—C9—C10—C11	−55.3 (5)	C10—C9—O1—C3	−47.4 (5)
N2—C9—C10—C7	−50.9 (4)	O3—C11—O4—C12	−6.2 (8)
O1—C9—C10—C7	69.5 (4)	C10—C11—O4—C12	171.1 (5)
C14—C9—C10—C7	−175.5 (4)	C13—C12—O4—C11	96.3 (7)
N1—C7—C10—C11	−59.5 (4)	C2—C1—O5—C15	−166.0 (5)
C4—C7—C10—C11	178.8 (3)	C6—C1—O5—C15	13.8 (8)

### Hydrogen-bond geometry (Å, º)

Cg1 is the centroid of the C1–C6 ring.

**Table:**

*D*—H···*A*	*D*—H	H···*A*	*D*···*A*	*D*—H···*A*
N1—H1···O2^i^	0.86	2.12	2.962 (5)	168
N2—H2*A*···O2^ii^	0.86	2.11	2.936 (4)	162
C14—H14*B*···*Cg*1^iii^	0.96	2.62	3.570 (6)	171

Symmetry codes: (i) −*x*+1, *y*+1/2, −*z*; (ii) −*x*+1, *y*−1/2, −*z*; (iii) −*x*+1, *y*−1/2, −*z*+1.
