# Supplementary figures and images for: Crystal structure of ethyl (2S)-9-meth­oxy-2-methyl-4-oxo-3,4,5,6-tetra­hydro-2H- 2,6-methano­benzo[g][1,3,5]oxa­diazocine-11-carboxyl­ate
Source: Acta Crystallogr E Crystallogr Commun. 2015 Jan 17;71(Pt 2):o117–8. doi: 10.1107/S2056989015000559 (PMC4384574; doi:10.1107/S2056989015000559)

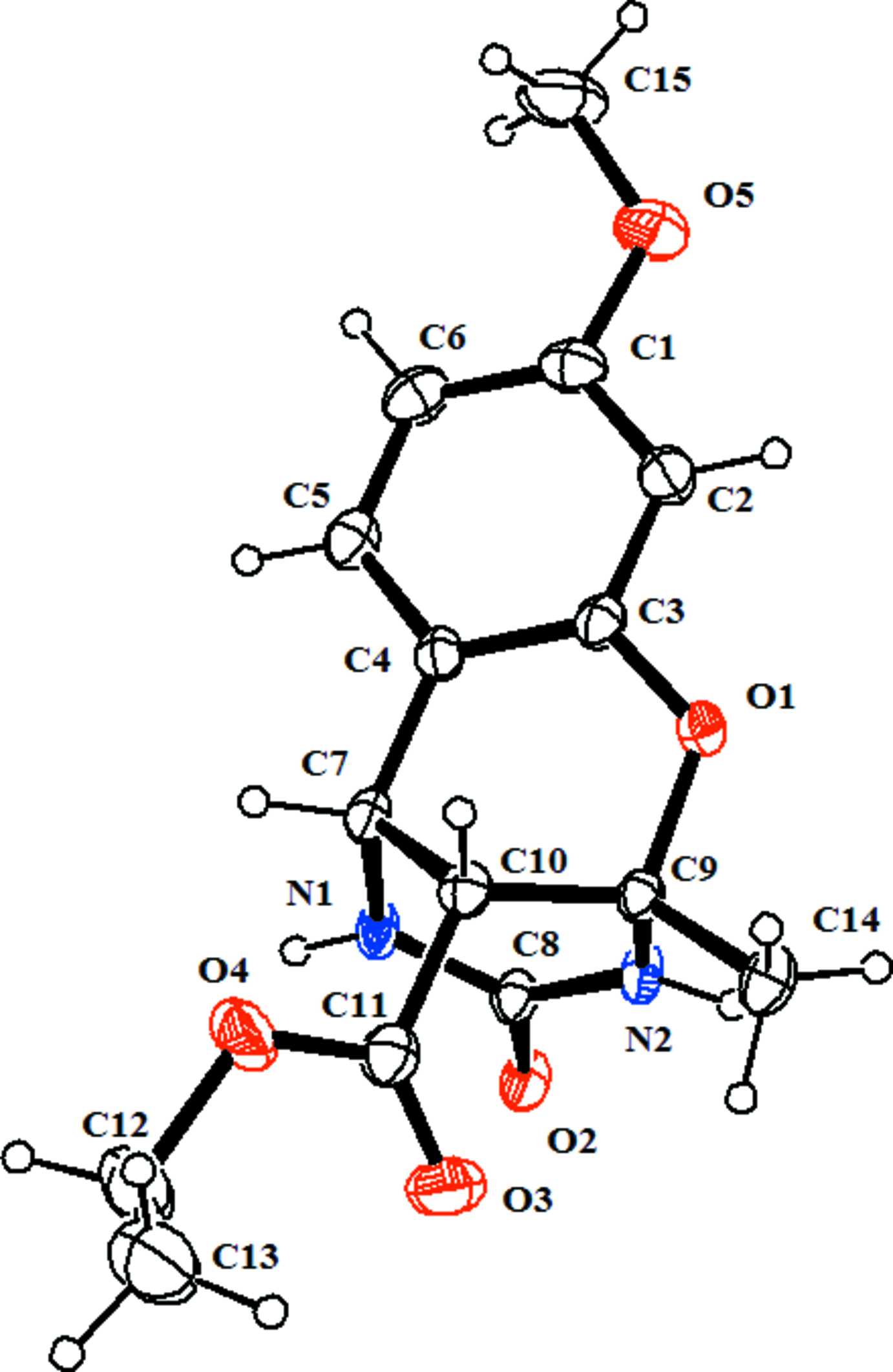

Supplement: Supplementary file 4 [file e-71-0o117-fig1.tif]

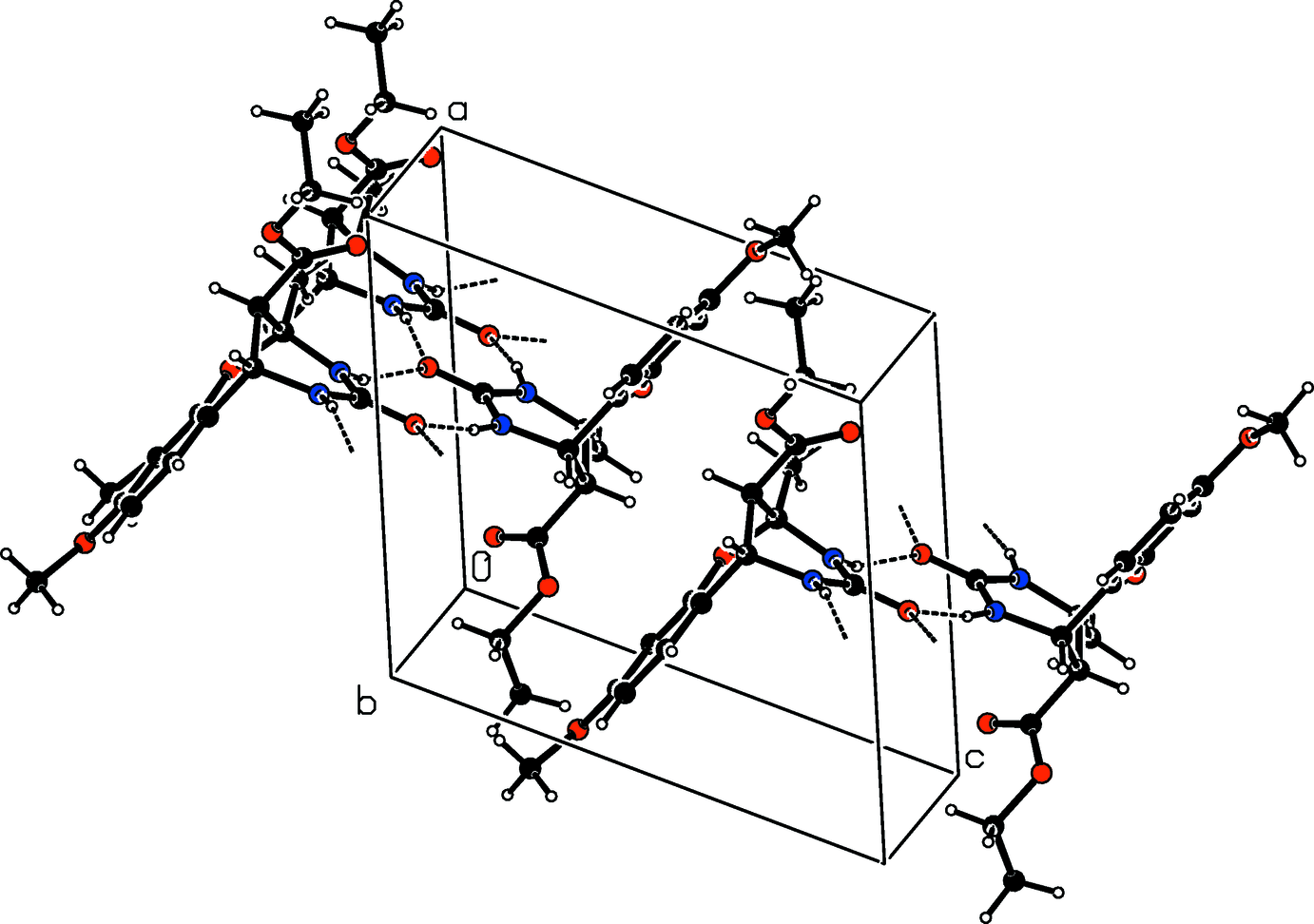

Supplement: Supplementary file 5 [file e-71-0o117-fig2.tif]
